# Carbonic Anhydrase in Pacific Abalone *Haliotis discus hannai*: Characterization, Expression, and Role in Biomineralization

**DOI:** 10.3389/fmolb.2021.655115

**Published:** 2021-04-15

**Authors:** Md. Rajib Sharker, Soo Cheol Kim, Shaharior Hossen, Kanij Rukshana Sumi, Sang Ki Choi, Kap Seong Choi, Kang Hee Kho

**Affiliations:** ^1^Department of Fisheries Science, College of Fisheries and Ocean Sciences, Chonnam National University, Yeosu, South Korea; ^2^Department of Fisheries Biology and Genetics, Faculty of Fisheries, Patuakhali Science and Technology University, Patuakhali, Bangladesh; ^3^Department of Aquaculture, Faculty of Fisheries, Patuakhali Science and Technology University, Patuakhali, Bangladesh; ^4^Department of Biological Sciences, College of Life Industry and Science, Sunchon National University, Suncheon, South Korea; ^5^Department of Food Science and Technology, Sunchon National University, Suncheon, South Korea

**Keywords:** *Haliotis discus hannai*, carbonic anhydrase, qRT-PCR, biomineralization, *in situ* hybridization

## Abstract

Carbonic anhydrases (CAs) are universal zinc ion containing metalloenzymes that play a pivotal role in various physiological processes. In this study, a CA I (designated as Hdh CA I) was isolated and characterized from the mantle tissue of Pacific abalone, *Haliotis discus hannai*. The full-length cDNA sequence of Hdh CA I was 1,417-bp in length, encoding a protein of 337 amino acids with molecular weight of 37.58 kDa. Hdh CA I sequence possessed a putative signal peptide of 22 amino acids and a CA catalytic function domain. The predicted protein shared 94 and 78% sequence identities with *Haliotis gigantea* and *Haliotis tuberculata* CA I, respectively. Results of phylogenetic analysis indicated that Hdh CA I was evolutionarily close to CA I of *H. gigantea* and *H. tuberculata* with high bootstrap values. Significantly higher levels of Hdh CA I mRNA transcript were found in mantle than other examined tissues. *In situ* hybridization results showed strong hybridization signals in epithelial cells of the dorsal mantle pallial, an area known to synthesize and secrete proteins responsible for the nacreous layer formation of shell. This is the first study on Hdh CA I in *H. discus hannai* and the results may contribute to further study its physiological functions in shell biomineralization of abalone.

## Introduction

Carbonic anhydrases (CAs) are ubiquitous zinc-binding metalloenzymes that regulate acid-base balance through catalyzing a simple physiological reaction: the conversion of CO_2_ to bicarbonate ions and protons (Lindskog and Silverman, 2000). CA reaction is involved in a vast range of physiological and pathological processes including respiration, acid-base balance, electrolyte secretion, calcification, signal transduction, oncogenesis, proliferation, and biosynthetic mechanisms (Chegwidden and Carter, 2000; Kuo et al., 2005; Thiry et al., 2006; Gilmour et al., 2007; Purkerson and Schwartz, 2007; Ditte et al., 2011; Brown et al., 2012; Frasseto et al., 2012). CA is indispensable for osmoregulation in fresh water and marine water species (Henry and Swenson, 2000; Perry and Gilmour, 2006). In mollusk, it has been postulated that CA is involved in shell formation by catalyzing the hydration of CO_2_ (Nielsen and Frieden, 1972).

There are seven evolutionary unrelated gene families of CAs in prokaryotes and eukaryotes, namely α-, β-, γ-, δ-, η-, ζ-, and θ-CAs (Krishnamurthy et al., 2008; Del Prete et al., 2015; Supuran and Capasso, 2015; Kikutani et al., 2016). α-CAs contain multiple isoforms of the enzyme and have been extensively studied in vertebrate and mammals (Aspatwar et al., 2010). In mammals, 16 different α-CA isoforms have been reported based on their catalytic efficacy, molecular signature, kinetic profiles, subcellular localization, and tissue distribution (Nishimori et al., 2007; Alterio et al., 2009). Eight cytosolic forms (CA I, CA II, CA III, CA VII, CA VIII, CA X, CA XI, and CA XIII), five membrane-bound isozymes (CA IV, CA IX, CA XII, CA XIV, and CA XV), two mitochondrial forms (CA VA, and CA VB), and a secreted CA have been reported up to date, exhibiting different tissue-specific expression, kinetic properties, and sensitivities to inhibitors notably sulfonamides and their derivatives (Supuran, 2008). CA VIII, X, and XI isoforms are designated as carbonic anhydrase related proteins (CARPs) owing to the lack of one or more Zn binding histidine residues at the active site (Aspatwar et al., 2010). Most CAs are characterized by the presence of a Zn^2+^ ion coordinated by three histidine residues except for those in the ζ-CA family, in which zinc is replaced by cadmium (Lane et al., 2005).

CA I is a cytoplasmic isozyme belonging to the α-CA family. It is involved in carbon dioxide transport, ion exchange, and acid-base balance. This isoenzyme was first identified and characterized from *Chlamydomonas reinhardtii*, encoding a polypeptide of 377 amino acid residues (Fujiwara et al., 1990). The common skeleton of carbonic anhydrase I of human contains an N-terminus active site, a zinc binding site, and a substrate-binding site. CA I is a low activity enzyme generally found in the cytosol of RBCs, gastrointestinal tract, and cardiac tissues of mammals (Chegwidden and Carter, 2000). It can induce retinal cerebral vascular permeability through prekallikrein activation and serine protease factor XII generation in human (Gao et al., 2007).

*Haliotis discus hannai* is an important commercial marine gastropod mollusk inhabiting in Japan, China, Taiwan, and Korean peninsula. *H. discus hannai* is considered a top-priced seafood item because it contains health beneficial bioactive molecules besides its basic nutritional value (Suleria et al., 2017). Many studies regarding cytosolic CA genes have been performed in different teleosts, non-teleosts, cyclostomes, and mammals (Murakami et al., 1987; Lund et al., 2002; Esbaugh et al., 2005, 2009; Gilmour et al., 2007; Sumi et al., 2019). Two CAs have been cloned in the mantle tissue of European abalone, *Haliotis tuberculata* (Le Roy et al., 2012). To date, characterization and expression profile of CA I in the Pacific abalone (*H. discus hannai*) have not been reported yet. Therefore, the objective of the present study was to isolate and molecularly characterize CA I gene from *H. discus hannai*, which will provide a unique insight to structural and signaling functions of this isoenzyme.

## Materials and Methods

### Experimental Animals and Tissue Collection

Three-year-old adult Pacific abalone (*H. discus hannai*) of both sexes with an average body weight of 128.2 ± 0.86 g and a shell length of 10.5 ± 0.12 cm were collected from Jindo Island, South Korea and transferred to the Department of Fisheries Science, Chonnam National University. Cerebral ganglion, mantle, gill, heart, shell muscle, hemocyte, and gonadal tissues (testis and ovary) were dissected, immediately frozen in liquid N_2_, and stored at −80°C for subsequent molecular analyses. Cryosection preparation from shell-forming mantle tissue was performed as described previously (Sharker et al., 2020a,b). All animal experiments were performed in accordance with guidelines of the Institutional Animal Care and Use Committee of Chonnam National University (approval number: CNU IACUC-YS-2020-5).

### RNA Isolation and cDNA Synthesis

Total RNAs were isolated from different tissues of Pacific abalone using an RNeasy mini kit (Qiagen, Hilden, Germany) according to the manufacturer’s protocol. RNA was treated with DNase I (Promega, Madison, WI, United States) to get rid of any genomic DNA contamination. Subsequently, 1 μg of total RNA was used for cDNA synthesis using a Superscript^®^ III First-Strand synthesis kit (Invitrogen, Carlsbad, CA, United States).

### Molecular Cloning and Sequencing of Full-Length cDNA in *H. discus hannai*

To perform molecular cloning, reverse transcription (RT) primers (sense: 5′-CATGGGTATGATGGACATTGC-3′; antisense: 5′-GATGGAGTTCAGCTCGAAGT-3′) were designed based on known *Haliotis gigantea* CA isozyme sequence (GenBank accession no. AB500103.2). RT-PCR was performed using Phusion^®^ High-Fidelity DNA Polymerase (Biolabs Inc., New England). The reaction mixture consisted of 1 μl synthesized cDNA template from mantle tissue, 1 μl (20 pmol) each of forward and reverse primers, 4 μl of 5× Phusion HF buffer (1×), 2 μl of dNTP (200 μM), 0.5 μl of 1 U Phusion DNA polymerase, and 10.5 μl sterile distilled water (dH_2_O) in a final volume of 20 μl. PCR reactions were performed under the following amplification conditions: 3 min at 95°C, followed by 35 cycles of 2 min at 94°C, 30 s at 56°C, 1 min at 72°C, with a final dissociation step at 72°C for 7 min. Amplified products were separated by 1.2% agarose gel electrophoresis and purified using a Wizard SV gel and PCR clean-up kit (Promega, Madison, WI, United States) following the manufacturer’s instruction. Subsequently, purified PCR fragments were ligated into pTOP Blunt V2 vector (Enzynomics, Daejeon, Korea) and transformed into competent *Escherichia coli* DH5α cells (Enzynomics). Plasmid DNA was then extracted from positive clones with a plasmid miniprep kit (Qiagen, Hilden, Germany) and sequenced using Macrogen Online Sequencing System (Macrogen, Seoul, Korea). To confirm the full-length sequence of CA I, 5′- and 3′-rapid amplification of cDNA ends (RACE) PCR was performed using a Smarter^®^ RACE 5′/3′ Kit (Clontech Laboratories, Inc., Mountain View, CA, United States) following the manufacturer’s protocol. Touchdown PCR was conducted with 30 cycles for 3′-RACE and 35 cycles for 5′-RACE using gene-specific primers (GSPs), including a 15-bp overlap with the 5′-end of the GSP sequence (antisense primer: 5′-GATTAC GCCAAGCTTGTGTCCACGTAGATCGGAGACTGGTTG-3′, sense primer: 5′-GATTACGCCAAGCTTCACCCTAAGCTG GCTGGACTTGATATCG-3′), a universal primer mix (UPM, 5′-CTAATACGACTCACTATAGGGCAAGCAGTGGTATCAACG CAGAGT-3′), and SeqAmp DNA polymerase in a total volume of 50 μl in accordance with kit instructions. NucleoSpin^®^ Gel and PCR Clean-Up kit was used to purify RACE PCR products. Subsequently, purified PCR products were ligated into linearized pRACE vector, transformed into Stellar Competent Cells, and then sequenced as described previously. These sequenced RACE products were then assembled by overlapping with the initial cloned cDNA fragment.

### Sequence and Phylogenetic Tree Analysis

*In silico* analysis of *H. discus hannai* CA I sequence was performed using multiple bioinformatics tools. Search for amino acid homology was performed with Basic Local Alignment Search Tool (BLASTP) against NCBI database^1^. SMART, a web-based tool^2^, was used for the identification and annotation of CA domain architecture. Expert protein analysis system^3^ was used to determine primary structures and subcellular localization of this gene. Multiple alignments of deduced amino acid sequences of CA isoforms were performed using Clustal Omega package (Sievers et al., 2011; Alva et al., 2016). Jalview Java alignment editor^4^ was used to edit and visualize multiple sequence alignments (Waterhouse et al., 2009). NetNGlyc 1.0 server^5^ and NetPhosK 3.1 server^6^ were used to predict potential N-linked glycosylation motif and serine/threonine phosphorylation sites, respectively. SignalP 4.1 (Petersen et al., 2011) was used to predict N-terminal signal peptide. Bonding state of cysteines in the protein sequence was predicted with CYSPRED (Fariselli et al., 1999). A phylogenetic tree was generated for orthologs of cytoplasmic CAs in vertebrates and invertebrates, and molluscan nacrein sequences. MEGA software (version 7.0) with a neighbor-joining (NJ) algorithm was employed to construct the phylogenetic tree (Kumar et al., 2016). Reliability of the tree was evaluated by bootstrapping using 1,000 bootstrap replications.

### Template Identification and Homology Modeling of CA I in *H. discus hannai*

Homology modeling of the three-dimensional (3D) protein structure of *H. discus hannai* CA I was performed using MODELLER^7^ by optimally satisfying spatial restraints (Šali and Blundell, 1993). The cloned full-length CA I sequence was subjected to a protein blast alignment against protein data bank (Berman et al., 2000)^8^. The crystal structure of human CA I (PDB ID: 1CZM) with a resolution of 2.00 Å was picked as a template to predict the 3D structure of cloned CA I. The best CA I model of *H. discus hannai* was selected based on normalized Discrete Optimized Protein Energy statistical score (zDOPE). Protein Quality Predictor (Wallner and Elofsson, 2003), Verify3D (Eisenberg et al., 1997), and ERRAT (Colovos and Yeates, 1993) tools were used to assess the stereochemical profile of the predicted model.

### Quantitative Real-Time PCR Expression Analysis

Gene-specific primer pairs (forward: 5′-GAGAAAACGC TACGATGCTG-3′ and reverse: 5′-GCTCTCCTTCACACAA TGG-3′) designed from cloned CA I sequence were used for quantitative real-time (qRT)-PCR assay. Ribosomal protein L-5 (RPL-5, GenBank accession no: JX002679.1) (forward: 5′-TGTCCGTTTCACCAACAAGG-3′ and reverse: 5′-AGATGG AATCAAGTTTCAATT-3′) as a reference gene was used for normalizing mRNA expression levels based on its expression stability (Wan et al., 2011). qPCR was performed using 2× qPCR BIO SyGreen Mix Lo-Rox kit in triplicates on a LightCycler^®^ 96 System (Roche, Germany) with a total reaction volume of 20 μl containing 10 μl of SyGreen mix, 1 μl of cDNA template from different tissues, 1 μl of each forward and reverse primer, and PCR-grade water to make up the volume. Three biological replicates (*N* = 3) were used for each tissue sample. Thermocycling parameters for PCR reactions were: 95°C for 3 min followed by 40 cycles of a three-step amplification at 94°C for 1 min, 60°C for 30 s, and 72°C for 1 min. Relative mRNA expression level was evaluated based on cycle threshold (Ct) using the 2^–ΔΔ*ct*^ method.

### *In Situ* Hybridization

DIG-labeled antisense and sense RNA probes were generated from the coding region of CA nucleotide sequence by *in vitro* transcription following a published procedure (Sharker et al., 2020c,d). Briefly, prehybridization of mantle tissue was performed with hybridization buffer and yeast total RNA for 2 h followed by hybridization with RNA probe at 65°C overnight. Subsequently, hybridized tissue sections were washed, incubated with a blocking solution at room temperature for 1 h and overnight at −20°C using an alkaline phosphatase-conjugated anti-digoxigenin antibody (diluted 1:2,000 in blocking solution) (Roche) to ascertain hybridization signals. These tissue sections were then treated with a labeling mix (2 ml of alkaline tris buffer, 9 μl of NBT, and 7 μl of BCIP) to visualize the color. To obtain the desired color, slides were washed with PBST, fixed with 4% PFA in PBS for 1 h, mounted with Permount mounting medium, covered with coverslips, and viewed with a stereo microscope (SMZ1500, Nikon, Tokyo, Japan).

### Statistical Analysis

Data were statistically analyzed using one-way analysis of variance (ANOVA) followed by Tukey’s multiple comparison test to identify differences among mean relative mRNA expression values in different tissues. All analyses were performed using SPSS version 16.0 (SPSS Inc., Chicago, IL, United States). Differences were considered statistically significant at *p* < 0.05.

## Results

### Cloning and Sequence Analysis of CA I in *H. discus hannai*

**FIGURE 1:**
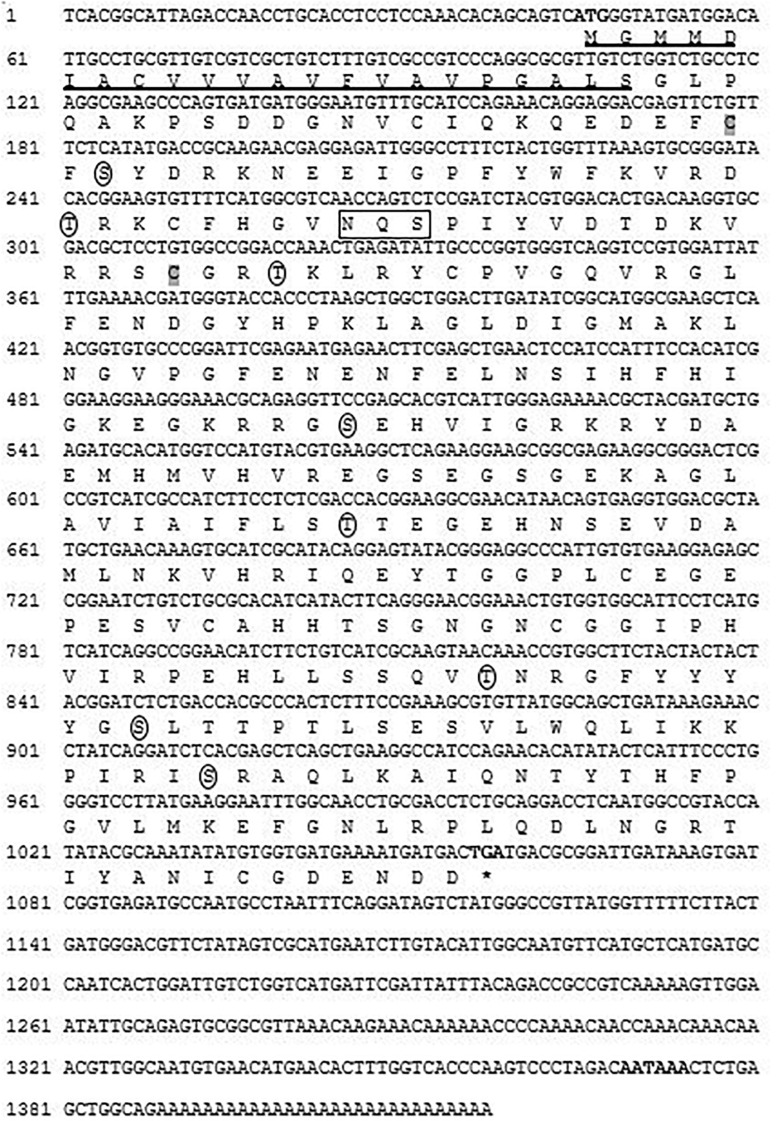
Nucleotide and deduced amino acid (one letter code) sequence of cloned CA I isozyme from *H. discus hannai*. Bold font indicates the initiation codon, termination codon (asterisks), and putative polyadenylation signal (AATAAA). The N-terminal signal peptide is underlined. A potential Asn-Xaa-Ser/Thr sequon is enclosed in a box. Circles indicate putative phosphorylation sites for protein kinase A or C. Two cysteine residues (Cys-45 and Cys-89) that are likely to form one intramolecular disulfide bond are shaded in gray.

Full-length cDNA sequence of CA I was successfully cloned from shell-forming mantle tissue of Pacific abalone, *H. discus hannai* (referred to as Hdh CA I). This sequence has been deposited at GenBank database under the accession number MT345603. It is 1,417-bp in length, including 5′- and 3′-untranslated region (UTR). It has an open reading frame of 1,014 nucleotides encoding a polypeptide of 337 amino acids with predicted molecular mass of 37.58 kDa and an isoelectric point (p*I*) of 6.54. The 3′-UTR had a canonical polyadenylation signal sequence (AATAAA) at 14-bp upstream of the poly-A tail. According to instability index, the deduced CA I gene in Pacific abalone was categorized as a stable protein. Domain architecture analysis revealed that Hdh CA I (from ^46^F to ^330^I) shared similarity with a potential α-CA isoform I. An NH_2_-terminal signal peptide of 22 amino acids in length was predicted, followed by a cleavage site between Ser^22^ and Gly^23^ residues (Figure 1).

NCBI BLASTP search revealed that the putative CA I shared 94 and 78% sequence identities with *H. gigantea* CA I and *H. tuberculata* CA I, respectively. Multiple sequence alignment output revealed that the protein sequence of Pacific abalone CA I shared 29, 27, and 26% identities with rainbow trout (*Oncorhynchus mykiss*, NP_001117692.1), human (*Homo sapiens*, P00915.2), and mouse (*Mus musculus*, NP_001077426.1) CA I, respectively. *In silico* analysis predicted that this protein might be a membrane bound extracellular protein. Its sequence possessed eight potential phosphorylation sites (at positions ^47^S, ^66^T, ^92^T, ^154^S, ^194^T, ^258^T, ^268^S, and^290^S) by protein kinase A or C. Two cysteine residues (Cys-45 and Cys-89) in this sequence seemed to be involved in forming an intrachain disulfide bridge. Analysis of its amino acid compositions revealed that glycine was the most abundant amino acid (10.7%), while tryptophan was the least abundant (0.6%) (Table 1).

**TABLE 1:** Sequence compositions of Pacific abalone CA I CD regions at amino acid levels.

Amino acid	% Composition
Alanine	4.7
Arginine	5.9
Asparagine	5.3
Aspartic acid	4.7
Cysteine	3
Glutamine	3.3
Glutamic acid	7.4
Glycine	10.7
Histidine	4.2
Isoleucine	5.6
Leucine	6.8
Lysine	5.3
Methionine	2.4
Phenylalanine	4.2
Proline	4.7
Serine	5.6
Threonine	4.2
Tryptophan	0.6
Tyrosine	3.9
Valine	7.4

**FIGURE 2:**
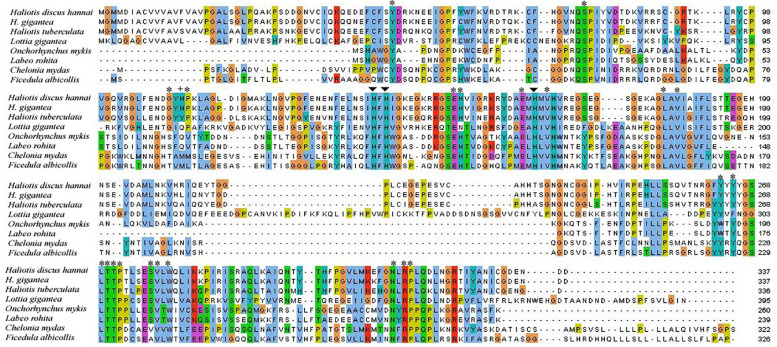
Alignment of CA I cloned from *H. discus hannai* and CA Is of other protostome and deuterostome species. Zinc-binding histidine residues and proton shuttling ligand are indicated by arrows and plus sign, respectively. Asterisks indicate residues that form the hydrogen bond of the CA I active site.

The potential CA domain of Pacific abalone was highly conserved with other CAs of vertebrates and invertebrates (Figure 2). Three histidine residues binding to zinc ion in the active site were highly conserved in all CA isoforms examined herein. Amino acids involved in the hydrogen bond network of the active site were also conserved among CAs of gastropod mollusk.

**FIGURE 3:**
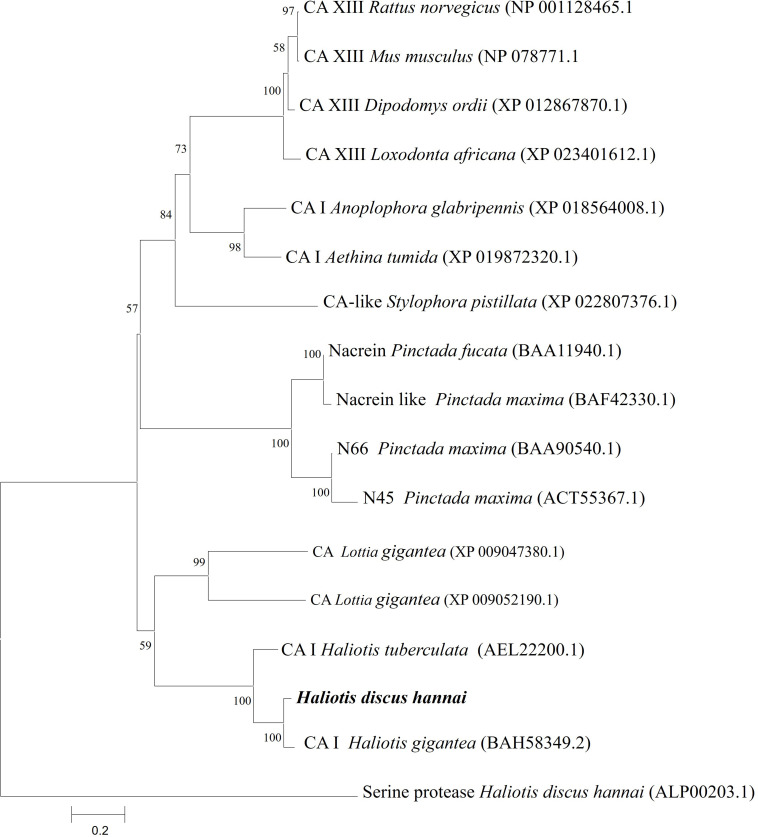
Phylogenetic tree based on amino acid sequences of Hdh CA I and other related proteins from vertebrates and invertebrates. A phylogenetic tree was constructed using the neighbor-joining method with 1,000 bootstrap replicates. The scale bar represents an evolutionary distance of 0.2 amino acid substitutions per site. GenBank accession numbers are shown in parentheses. Hdh CA I is highlighted in bold font. *H. discus hannai* serine protease was used as an outgroup.

A phylogenetic tree was constructed using representative species of vertebrates and invertebrates CAs along with nacreins of molluscans using the NJ method. The phylogenetic tree revealed several clades. The CA I of *H. discus hannai* was clustered with gastropod clade and properly aligned with CA I of *H. gigantea* and *H. tuberculata* (Figure 3).

**FIGURE 4:**
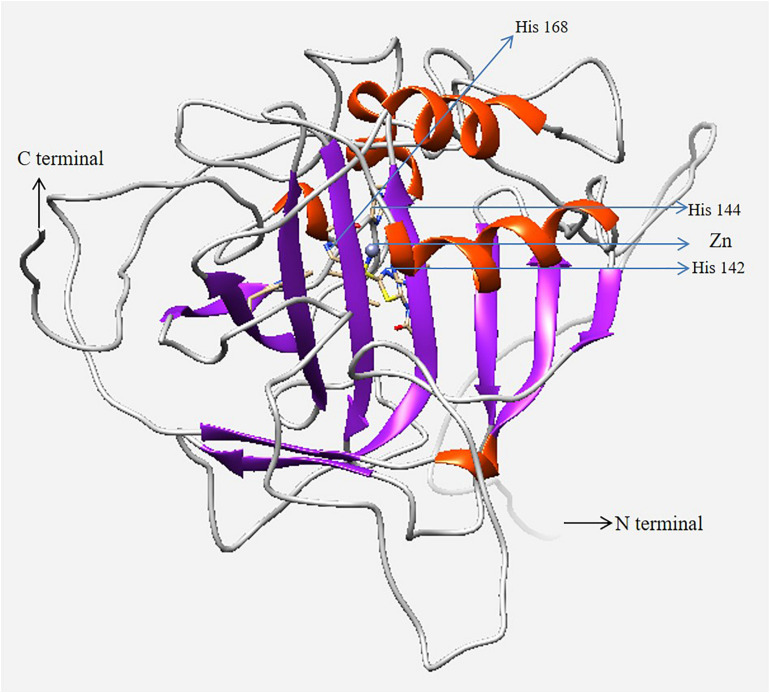
Three-dimensional homology model of CA I isolated from *H. discus hannai*. N- and C-termini are marked with black arrows. The zinc ion in the active site and coordinated three histidine residues are marked in the figure with blue arrows. The model was generated using UCSF Chimera software.

According to the similarity index of several amino acid signature, human CA I (PDB 1CZM) was employed as a template to predict the 3D structure of *H. discus hannai* CA I (Figure 4). Validation results (LG score of 1.780, value > 1.5 indicating fairly good model) revealed that the predicted model was fairly good. The Verify3D: 3D/1D profile score was 87.53% and the ERRAT quality factor for the predicted CA I protein was 82.94%.

### Tissue Expression Analysis of CA I in Pacific Abalone

**FIGURE 5:**
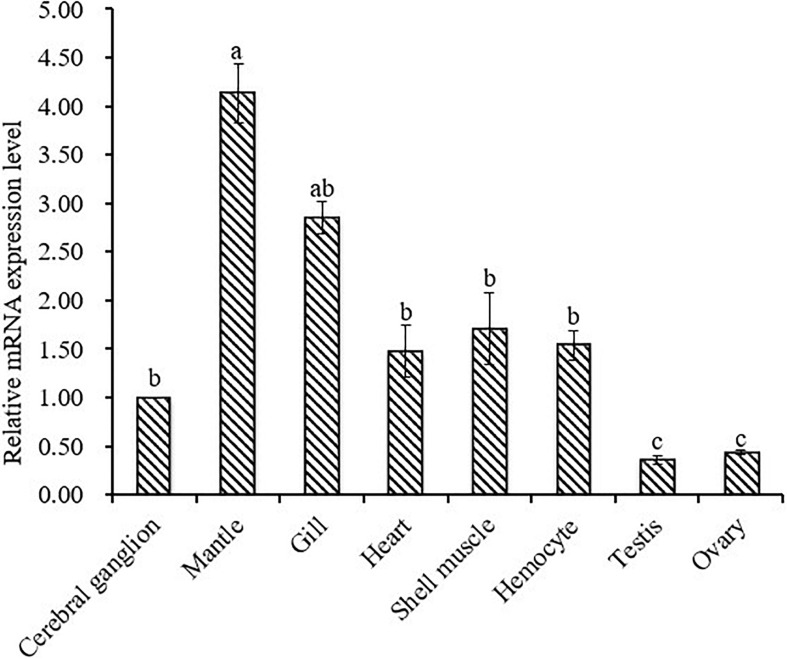
Expression of Hdh CA I mRNA (mean ± SD, *N* = 3) in various tissues of *H. discus hannai* detected by qRT-PCR. The expression level in the cerebral ganglion is set as 1.00 to calibrate relative levels of mRNA in other examined tissues. Means not sharing the same superscripts are significantly (*p* < 0.05) different from each other.

Relative mRNA expression levels of CA I in different tissues were determined by qRT-PCR assay. Results of analysis revealed that Hdh CA I mRNA was expressed predominantly in the mantle, showing moderate levels in the gill, cerebral ganglion, heart, shell muscle, and hemocyte. However, its expression levels were low in testis and ovary. As shown in Figure 5, relative mRNA expression levels of Hdh CA I were significantly (*p* < 0.05) higher in mantle than in other examined tissues.

**FIGURE 6:**
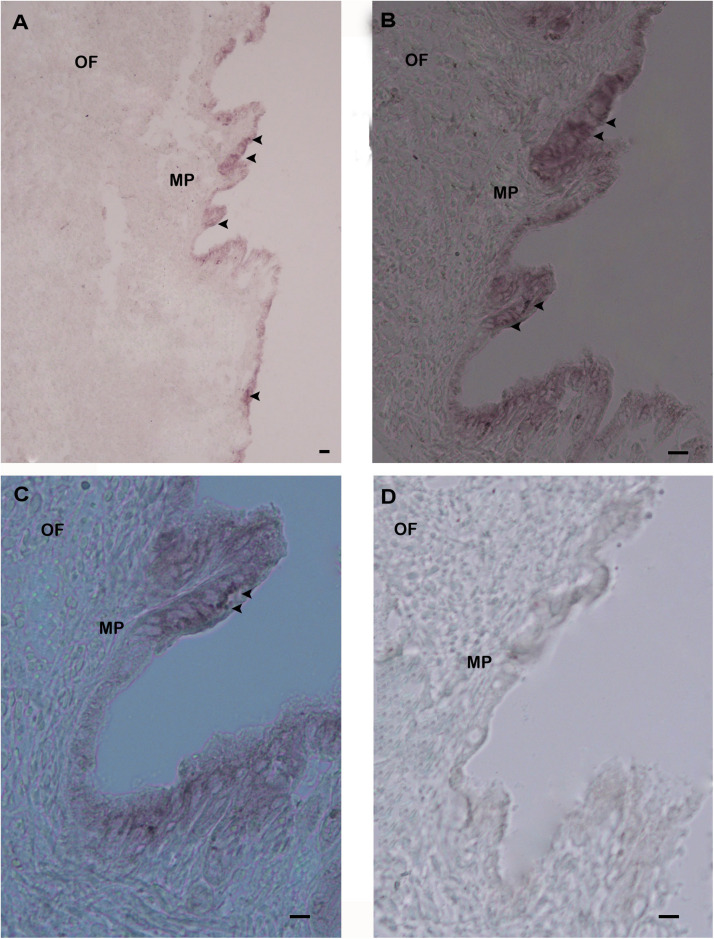
*In situ* hybridization analysis of Hdh CA I mRNA expression in the mantle of *H. discus hannai*. **(A)** Positive hybridization signals with an antisense probe were detected in the outer epithelium of mantle pallial edge. **(B)** Medium magnification of **(A)**. **(C)** High magnification showing hybridized CA I mRNA in mantle pallial (MP) edge. **(D)** Negative control with a sense probe showed no hybridization signal. Strong hybridization signals are indicated by black arrowheads. Scale bar, 100 μm. OF, outer fold; MP, mantle pallial.

To explore the functional role of Hdh CA I in shell formation, cellular localization of its mRNA was demonstrated by *in situ* hybridization (ISH) using mantle tissue sections. Positive signals of Hdh CA I mRNA were found in epithelial cells of the dorsal mantle pallial, a region known to express genes involved in the biosynthesis of the nacreous layer of the shell (Figures 6A–C). However, hybridization with a sense probe (negative control) showed no hybridization signal (Figure 6D).

## Discussion

From protostomes to deuterostomes, CAs plays a critical role in various aspects of physiological processes (Chegwidden and Carter, 2000). Several studies have been conducted on CA isozyme in bivalve and gastropod mollusk (Le Roy et al., 2012; Ip et al., 2017; Perfetto et al., 2017). However, characterization and expression of CA in shell forming mantle tissues of Pacific abalone have not been reported yet. In this study, the full-length sequence of CA isozyme in *H. discus hannai* was obtained and biomolecular characteristics of this protein with its mRNA expression profile were analyzed. A previous study on nacrein in *Pinctada fucata* showed low catalytic activity than CA activity (Miyamoto et al., 1996). Nacrein contain GXN repeat domain possibly binds calcium for participating calcium carbonate crystal formation of the nacreous layer. In the present study, sequence analysis indicates that Hdh CA I do not possess GXN repeat domain and therefore our reported CA is truly α-CA. A characteristic signal peptide of 22 amino acids was found in this cloned sequence, indicated that Hdh CA I was an extracellularly secreted or membrane bound protein. Wang et al. (2017) have reported a secreted or membrane bound α-CA in bivalve mollusk, *Crassostrea gigas*. Several key motifs including phosphorylation sites and N-linked glycosylation sites were also identified in Hdh CA I, consistent with previous studies (Le Roy et al., 2012; Song et al., 2014). Phosphorylation site plays a crucial role in various signal transduction pathways (Ali et al., 2016). It has been reported that phosphorylation of amino acid may contribute to the formation or accumulation of minerals by interacting with Ca^2+^ (Song et al., 2014). Two cysteine residues in Hdh CA I were predicted to form an intrachain disulfide link known to be crucial for protein structure stabilizing and maintaining biological functions of this protein (Kadokura et al., 2004; Inaba et al., 2006). Sequence alignment revealed that Hdh CA I shared high identities with the catalytic domain of CAs in different vertebrates and invertebrates (Figure 2). Three histidine residues that could bind with a catalytic Zn^2+^ were also conserved in the CA I of *H. discus hannai*. The architectural domain of Hdh CA I contained a proton shuttling residue, a substrate associated pocket, and a Thr-199 loop site known to be essential for the catalytic activity of this isozyme (Esbaugh and Tufts, 2006). Hydrophobic residues known to be crucial for CO_2_ substrate binding were conserved in all CAs analyzed in this study.

In the phylogenetic tree, Hdh CA I gene was phylogenetically grouped with *H. gigantea* CA I (Figure 3). According to phylogenetic analysis, the Hdh CA I clade has diverged from CAs of vertebrates and nacreins protein. Previous studies have shown that CA I of *H. tuberculata* is clustered with CA I isoform of *H. gigantea* (Le Roy et al., 2012).

To predict the 3D structure of Hdh CA I, the crystal structure of human CA I with a resolution of 2.00 Å was used as template (Figure 4). Zn-bound hydroxide ions are known to play pivotal role in enzymatic activities of CA proteins (Lindskog and Silverman, 2000). Thus, Zn^2+^ was considered during homology analysis in the present study. Evaluation results confirmed that the cloned Hdh CA I protein in favorable positions for each amino acid residue.

Hdh CA I mRNA transcript was found in all tested tissues, with mantle showing significantly higher expression (Figure 5). Tissue expression analysis agreed with previous results (Le Roy et al., 2012; Ip et al., 2017), suggesting that mantle is the principal site of CA I expression. Results of analysis also suggested that Hdh CA I might play a crucial role in the shell formation process. Rousseau et al. (2003) have reported that mantle is the most important calcium compartmental tissue because it is actively involved in shell biomineralization. Relatively higher expression levels of Hdh CA I in gill indicate that the involvement of this gene in respiration, pH regulation, ion transport and modulates of ionic concentration. Previous studies showed CA activity in gill of *C. gigas* and *H. tuberculata* (Duvail and Fouchereau-Peron, 2001; Wang et al., 2017).

Previous studies have reported that matrix proteins are expressed and secreted from various parts of mantles and play key roles in shell biomineralization (Liu et al., 2011). Mantle pallial of oyster expresses CA mRNA which plays an important role in nacreous layer formation (Miyamoto et al., 1996). In the present study, ISH experiment demonstrated that the outer epithelium of the mantle pallial showed strong hybridization signals of Hdh CA I mRNA, suggesting that this gene might be involved in the formation of the nacreous layer for shell mineralization.

## Conclusion

In summary, a secreted α-type CA was identified from *H. discus hannai* for the first time, and its mRNA levels in different tissues were analyzed. Significantly upregulated expression of Hdh CA I was detected in mantle. ISH results demonstrated that Hdh CA I mRNA was specifically expressed in epithelial cells of the dorsal mantle pallial, suggesting that this gene might be responsible for shell biomineralization by regulating CaCO_3_ crystal formation of the nacreous layer.

## Data Availability Statement

The datasets presented in this study can be found in online repositories. The names of the repository/repositories and accession number(s) can be found in the article/supplementary material.

## Ethics Statement

The animal study was reviewed and approved by all animal experiments were performed in accordance with guidelines of the Institutional Animal Care and Use Committee of Chonnam National University (approval number: CNU IACUC-YS-2020-5).

## Author Contributions

KK designed the experiments and prepared the manuscript. MS designed and conducted all the experiment, analyzed the data, and wrote the manuscript. SK and SH conducted the *in situ* hybridization and qPCR experiment. KS performed bioinformatics analysis. SC and KC revised the manuscript and gave intellectual input to improve it. All authors read and approved the final manuscript.

## Conflict of Interest

The authors declare that the research was conducted in the absence of any commercial or financial relationships that could be construed as a potential conflict of interest.

## Footnotes

1 http://www.ncbi.nlm.nih.gov/BLSAT/

2 http://smart.embl-heidelberg.de

3 https://www.expasy.org/

4 www.jalview.org

5 http://www.cbs.dtu.dk/services/NetNGlyc/

6 http://www.cbs.dtu.dk/services/NetPhos/

7 https://salilab.org/modeller/

8 www.rcsb.org

## References

[B1] Ali E. S. Hua J. Wilson C. H. Tallis G. A. Zhou F. H. Rychkov G. Y. (2016). The glucagon-like peptide-1 analogue exendin-4 reverses impaired intracellular Ca2 + signalling in steatotic hepatocytes. *Biochim. Biophys. Acta Mol. Cell Res.* 1863 2135–2146. 10.1016/j.bbamcr.2016.05.006 27178543

[B2] Alterio V. Hilvo M. Di Fiore A. Supuran C. T. Pan P. Parkkila S. (2009). Crystal structure of the catalytic domain of ythe tumor-associated human carbonic anhydrase IX. *Proc. Natl. Acad. Sci. U.S.A.* 106 16233–16238. 10.1073/pnas.0908301106 19805286 PMC2752527

[B3] Alva V. Nam S.-Z. Söding J. Lupas A. N. (2016). The MPI bioinformatics Toolkit as an integrative platform for advanced protein sequence and structure analysis. *Nucleic Acids Res.* 44 W410–W415. 10.1093/nar/gkw348 27131380 PMC4987908

[B4] Aspatwar A. Tolvanen M. E. E. Parkkila S. (2010). Phylogeny and expression of carbonic anhydrase-related proteins. *BMC Mol. Biol.* 11:25. 10.1186/1471-2199-11-25 20356370 PMC2873310

[B5] Berman H. M. Westbrook J. Feng Z. Gilliland G. Bhat T. N. Weissig H. (2000). The protein data bank. *Nucleic Acids Res.* 28 235–242. 10.1093/nar/28.1.235 10592235 PMC102472

[B6] Brown B. F. Quon A. Dyck J. R. B. Casey J. R. (2012). Carbonic anhydrase II promotes cardiomyocyte hypertrophy. *Can. J. Physiol. Pharmacol.* 90 1599–1610. 10.1139/y2012-142 23210439

[B7] Chegwidden W. R. Carter N. D. (2000). “Introduction to the carbonic anhydrases,” in *The Carbonic Anhydrases. EXS 90*, Vol. 90 eds Chegwidden W. R. Carter N. D. Edwards Y. H. (Basel: Birkhäuser), 14–28. 10.1007/978-3-0348-8446-4_2 11268513

[B8] Colovos C. Yeates T. O. (1993). Verification of protein structures: patterns of nonbonded atomic interactions. *Protein Sci.* 2 1511–1519. 10.1002/pro.5560020916 8401235 PMC2142462

[B9] Del Prete S. Vullo D. De Luca V. Alothman Z. Osman S. M. Supuran C. T. (2015). Biochemical characterization of recombinant β-carbonic anhydrase (PgiCAb) identified in the genome of the oral pathogenic bacterium *Porphyromonas gingivalis*. *J. Enzyme Inhib. Med. Chem.* 30 366–370. 10.3109/14756366.2014.931383 25032746

[B10] Ditte P. Dequiedt F. Svastova E. Hulikova A. Ohradanova-Repic A. Zatovicova M. (2011). Phosphorylation of carbonic anhydrase IX controls its ability to mediate extracellular acidification in hypoxic tumors. *Cancer Res.* 71 7558–7567. 10.1158/0008-5472.CAN-11-2520 22037869

[B11] Duvail L. Fouchereau-Peron M. (2001). Calcium metabolism related markers during the growth of *Haliotis tuberculata*. *Invertebr. Reprod. Dev.* 40 209–216. 10.1080/07924259.2001.9652720

[B12] Eisenberg D. Lüthy R. Bowie J. U. (1997). VERIFY3D: assessment of protein models with three-dimensional profiles. *Methods Enzymol.* 277 396–404. 10.1016/S0076-6879(97)77022-8 9379925

[B13] Esbaugh A. J. Gilmour K. M. Perry S. F. (2009). Membrane-associated carbonic anhydrase in the respiratory system of the Pacific hagfish (*Eptatretus stouti*). *Respir. Physiol. Neurobiol.* 166 107–116. 10.1016/j.resp.2009.02.005 19429527

[B14] Esbaugh A. J. Perry S. F. Bayaa M. Georgalis T. Nickerson J. Tufts B. L. (2005). Cytoplasmic carbonic anhydrase isozymes in rainbow trout *Oncorhynchus mykiss*: comparative physiology and molecular evolution. *J. Exp. Biol.* 208 1951–1961. 10.1242/jeb.01551 15879075

[B15] Esbaugh A. J. Tufts B. L. (2006). The structure and function of carbonic anhydrase isozymes in the respiratory system of vertebrates. *Respir. Physiol. Neurobiol.* 154 185–198. 10.1016/j.resp.2006.03.007 16679072

[B16] Fariselli P. Riccobelli P. Casadio R. (1999). Role of evolutionary information in predicting the disulfide-bonding state of cysteine in proteins. *Proteins Struct. Funct. Genet.* 36 340–346. 10.1002/(SICI)1097-0134(19990815)36:3<340:AID-PROT8>3.0.CO;2-D 10409827

[B17] Frasseto F. Parisotto T. M. Peres R. C. R. Marques M. R. Line S. R. P. Nobre Dos Santos M. (2012). Relationship among salivary carbonic anhydrase vi activity and flow rate, biofilm ph and caries in primary dentition. *Caries Res.* 46 194–200. 10.1159/000337275 22508543

[B18] Fujiwara S. Fukuzawa H. Tachiki A. Miyachi S. (1990). Structure and differential expression of two genes encoding carbonic anhydrase in *Chlamydomonas reinhardtii*. *Proc. Natl. Acad. Sci. U.S.A.* 87 9779–9783. 10.1073/pnas.87.24.9779 2124702 PMC55257

[B19] Gao B. B. Clermont A. Rook S. Fonda S. J. Srinivasan V. J. Wojtkowski M. (2007). Extracellular carbonic anhydrase mediates hemorrhagic retinal and cerebral vascular permeability through prekallikrein activation. *Nat. Med.* 13 181–188. 10.1038/nm1534 17259996

[B20] Gilmour K. M. Bayaa M. Kenney L. McNeill B. Perry S. F. (2007). Type IV carbonic anhydrase is present in the gills of spiny dogfish (*Squalus acanthias*). *Am. J. Physiol. - Regul. Integr. Comp. Physiol.* 292 R556–R567. 10.1152/ajpregu.00477.2006 16973930

[B21] Henry R. P. Swenson E. R. (2000). The distribution and physiological significance of carbonic anhydrase in vertebrate gas exchange organs. *Respir. Physiol.* 121 1–12. 10.1016/S0034-5687(00)00110-9 10854618

[B22] Inaba K. Murakami S. Suzuki M. Nakagawa A. Yamashita E. Okada K. (2006). Crystal STRUCTURE of the DsbB-DsbA complex reveals a mechanism of disulfide bond generation. *Cell* 127 789–801. 10.1016/j.cell.2006.10.034 17110337

[B23] Ip Y. K. Koh C. Z. Y. Hiong K. C. Choo C. Y. L. Boo M. V. Wong W. P. (2017). Carbonic anhydrase 2-like in the giant clam, *Tridacna squamosa*: characterization, localization, response to light, and possible role in the transport of inorganic carbon from the host to its symbionts. *Physiol. Rep.* 5 1–15. 10.14814/phy2.13494 29199178 PMC5727267

[B24] Kadokura H. Tian H. Zander T. Bardwell J. C. A. Beckwith J. (2004). Snapshots of DsbA in action: detection of proteins in the process of oxidative folding. *Science* 303 534–537. 10.1126/science.1091724 14739460

[B25] Kikutani S. Nakajima K. Nagasato C. Tsuji Y. Miyatake A. Matsuda Y. (2016). Thylakoid luminal θ-carbonic anhydrase critical for growth and photosynthesis in the marine diatom *Phaeodactylum tricornutum*. *Proc. Natl. Acad. Sci. U.S.A.* 113 9828–9833. 10.1073/pnas.1603112113 27531955 PMC5024579

[B26] Krishnamurthy V. M. Kaufman G. K. Urbach A. R. Gitlin I. Gudiksen K. L. Weibel D. B. (2008). Carbonic anhydrase as a model for biophysical and physical-organic studies of proteins and protein-ligand binding. *Chem. Rev.* 108 946–1051. 10.1021/cr050262p 18335973 PMC2740730

[B27] Kumar S. Stecher G. Tamura K. (2016). MEGA7: molecular evolutionary genetics analysis Version 7.0 for bigger datasets. *Mol. Biol. Evol.* 33 1870–1874. 10.1093/molbev/msw054 27004904 PMC8210823

[B28] Kuo W. H. Yang S. F. Hsieh Y. S. Tsai C. S. Hwang W. L. Chu S. C. (2005). Differential expression of carbonic anhydrase isoenzymes in various types of anemia. *Clin. Chim. Acta* 351 79–86. 10.1016/j.cccn.2004.07.009 15563874

[B29] Lane T. W. Saito M. A. George G. N. Pickering I. J. Prince R. C. Morel F. M. M. (2005). A cadmium enzyme from a marine diatom. *Nature* 435:42. 10.1038/435042a 15875011

[B30] Le Roy N. Marie B. Gaume B. Guichard N. Delgado S. Zanella-Cléon I. (2012). Identification of two carbonic anhydrases in the mantle of the European abalone *Haliotis tuberculata* (*Gastropoda*, *Haliotidae*): phylogenetic implications. *J. Exp. Zool. Part B Mol. Dev. Evol.* 318 353–367. 10.1002/jez.b.22452 22711568

[B31] Lindskog S. Silverman D. N. (2000). The carbonic anhydrases new horizons: the catalytic mechanism of mammalian carbonic anhydrases. *EXS* 90 175–195. 10.1007/978-3-0348-8446-4_10 11268516

[B32] Liu X. Liu C. Chen L. Sun J. Zhou Y. Li Q. (2011). A new method to extract matrix proteins directly from the secretion of the mollusk mantle and the role of these proteins in shell biomineralization. *Mar. Biotechnol.* 13 981–991. 10.1007/s10126-011-9362-y 21279408

[B33] Lund S. G. Dyment P. Gervais M. R. Moyes C. D. Tufts B. L. (2002). Characterization of erythrocyte carbonic anhydrase in an ancient fish, the longnose gar (*Lepisosteus osseus*). *J. Comp. Physiol. B Biochem. Syst. Environ. Physiol.* 172 467–476. 10.1007/s00360-002-0269-9 12192508

[B34] Miyamoto H. Miyashita T. Okushimat M. Nakanoi S. Morita T. Matsushiro A. (1996). A carbonic anhydrase from the nacreous layer in oyster pearls. *Proc. Natl. Acad. Sci. U.S.A.* 93 9657–9660. 8790386 10.1073/pnas.93.18.9657 PMC38484

[B35] Murakami H. Marelich G. P. Grubb J. H. Kyle J. W. Sly W. S. (1987). Cloning, expression, and sequence homologies of cDNA for human carbonic anhydrase II. *Genomics* 1 159–166. 10.1016/0888-7543(87)90008-5 3121496

[B36] Nielsen S. A. Frieden E. (1972). Carbonic anhydrase activity in molluscs. *Comp. Biochem. Physiol. Part B Biochem.* 41 461–468. 10.1016/0305-0491(72)90107-1 4623971

[B37] Nishimori I. Minakuchi T. Onishi S. Vullo D. Cecchi A. Scozzafava A. (2007). Carbonic anhydrase inhibitors: cloning, characterization, and inhibition studies of the cytosolic isozyme III with sulfonamides. *Bioorganic Med. Chem.* 15 7229–7236. 10.1016/j.bmc.2007.08.037 17826101

[B38] Perfetto R. Del Prete S. Vullo D. Carginale V. Sansone G. Barone C. M. A. (2017). Cloning, expression and purification of the α-carbonic anhydrase from the mantle of the Mediterranean mussel, *Mytilus galloprovincialis*. *J. Enzyme Inhib. Med. Chem.* 32 1029–1035. 10.1080/14756366.2017.1353502 28741386 PMC6010101

[B39] Perry S. F. Gilmour K. M. (2006). Acid-base balance and CO_2_ excretion in fish: unanswered questions and emerging models. *Respir. Physiol. Neurobiol.* 154 199–215. 10.1016/j.resp.2006.04.010 16777496

[B40] Petersen T. N. Brunak S. Von Heijne G. Nielsen H. (2011). SignalP 4.0: discriminating signal peptides from transmembrane regions. *Nat. Methods* 8 785–786. 10.1038/nmeth.1701 21959131

[B41] Purkerson J. M. Schwartz G. J. (2007). The role of carbonic anhydrases in renal physiology. *Kidney Int.* 71 103–115. 10.1038/sj.ki.5002020 17164835

[B42] Rousseau M. Plouguerné E. Wan G. Wan R. Lopez E. Fouchereau-Peron M. (2003). Biomineralisation markers during a phase of active growth in *Pinctada margaritifera*. *Comp. Biochem. Physiol. A Mol. Integr. Physiol.* 135 271–278. 10.1016/S1095-6433(03)00070-9 12781827

[B43] Šali A. Blundell T. L. (1993). Comparative protein modelling by satisfaction of spatial restraints. *J. Mol. Biol.* 234 779–815. 10.1006/jmbi.1993.1626 8254673

[B44] Sharker M. R. Kim S. C. Sumi K. R. Sukhan Z. P. Sohn Y. C. Lee W. K. (2020a). Characterization and expression analysis of a GnRH-like peptide in the Pacific abalone, *Haliotis discus* hannai. *Agri Gene* 15 1–10. 10.1016/j.aggene.2019.100099

[B45] Sharker M. R. Nou I. S. Kho K. H. (2020b). Molecular characterization and spatiotemporal expression of prohormone convertase 2 in the Pacific abalone, *Haliotis discus* hannai. *PLoS One* 15:e0231353. 10.1371/journal.pone.0231353 32271824 PMC7144994

[B46] Sharker M. R. Sukhan Z. P. Kim S. C. Lee W. K. Kho K. H. (2020c). Identification, characterization, and expression analysis of a serotonin receptor involved in the reproductive process of the Pacific abalone, *Haliotis discus* hannai. *Mol. Biol. Rep.* 47 555–567. 10.1007/s11033-019-05162-2 31696430

[B47] Sharker M. R. Sukhan Z. P. Kim S. C. Lee W. K. Kho K. H. (2020d). Molecular identification, characterization, and expression analysis of a gonadotropin-releasing hormone Receptor (GnRH-R) in Pacific Abalone, *Haliotis discus* hannai. *Molecules* 25 1–15. 10.3390/molecules25122733 32545589 PMC7355911

[B48] Sievers F. Wilm A. Dineen D. Gibson T. J. Karplus K. Li W. (2011). Fast, scalable generation of high-quality protein multiple sequence alignments using Clustal Omega. *Mol. Syst. Biol.* 7:539. 10.1038/msb.2011.75 21988835 PMC3261699

[B49] Song X. Wang X. Li L. Zhang G. (2014). Identification two novel nacrein-like proteins involved in the shell formation of the Pacific oyster *Crassostrea gigas*. *Mol. Biol. Rep.* 41 4273–4278. 10.1007/s11033-014-3298-z 24584662 PMC4066178

[B50] Suleria H. A. R. Masci P. P. Gobe G. C. Osborne S. A. (2017). Therapeutic potential of abalone and status of bioactive molecules: a comprehensive review. *Crit. Rev. Food Sci. Nutr.* 57 1742–1748. 10.1080/10408398.2015.1031726 26114550

[B51] Sumi K. R. Kim S. C. Howlader J. Sharker M. R. Choi K. S. Choi S. K. (2019). Molecular identification and expression analysis of carbonic anhydrase VII in Pufferfish (*Takifugu rubripes*). *Ocean Sci. J.* 54 363–374. 10.1007/s12601-019-0020-z

[B52] Supuran C. T. (2008). Carbonic anhydrases: novel therapeutic applications for inhibitors and activators. *Nat. Rev. Drug Discov.* 7 168–181. 10.1038/nrd2467 18167490

[B53] Supuran C. T. Capasso C. (2015). The η-class carbonic anhydrases as drug targets for antimalarial agents. *Expert Opin. Ther. Targets* 19 551–563. 10.1517/14728222.2014.991312 25495426

[B54] Thiry A. Dogné J. M. Masereel B. Supuran C. T. (2006). Targeting tumor-associated carbonic anhydrase IX in cancer therapy. *Trends Pharmacol. Sci.* 27 566–573. 10.1016/j.tips.2006.09.002 16996620

[B55] Wallner B. Elofsson A. (2003). Can correct protein models be identified? *Protein Sci.* 12 1073–1086. 10.1110/ps.0236803 12717029 PMC2323877

[B56] Wan Q. Whang I. Choi C. Y. Lee J. S. Lee J. (2011). Validation of housekeeping genes as internal controls for studying biomarkers of endocrine-disrupting chemicals in disk abalone by real-time PCR. *Comp. Biochem. Physiol. C Toxicol. Pharmacol.* 153 259–268. 10.1016/j.cbpc.2010.11.009 21168524

[B57] Wang X. Wang M. Jia Z. Qiu L. Wang L. Zhang A. (2017). A carbonic anhydrase serves as an important acid-base regulator in pacific oyster *Crassostrea gigas* exposed to elevated CO2: implication for physiological responses of mollusk to ocean acidification. *Mar. Biotechnol.* 19 22–35. 10.1007/s10126-017-9734-z 28204970

[B58] Waterhouse A. M. Procter J. B. Martin D. M. A. Clamp M. Barton G. J. (2009). Jalview Version 2-A multiple sequence alignment editor and analysis workbench. *Bioinformatics* 25 1189–1191. 10.1093/bioinformatics/btp033 19151095 PMC2672624

